# Alternative management of proximal aortic dissection: concept and application

**DOI:** 10.1007/s12055-021-01281-3

**Published:** 2021-12-13

**Authors:** Xun Yuan, Andreas Mitsis, David Mozalbat, Christoph A. Nienaber

**Affiliations:** 1 Cardiology and Aortic Centre, Royal Brompton and Harefield Hospitals, Guy’s and St Thomas’ NHS Foundation Trust, London, SW3 6NP UK; 2 National Heart and Lung Institute, Faculty of Medicine, Imperial College London, London, UK; 3 Cardiology Department, Nicosia General Hospital, Strovolos, Cyprus; 4 Cardio-Thoracic Surgery Department, St George Hospital London, London, UK

**Keywords:** Proximal aortic dissection, TEVAR, Endovascular, Occluder, Coils

## Abstract

Open surgery remains the mainstay of treatment for acute type A aortic dissection and should be offered to most patients. However, there are elderly patients in which surgical treatment may be deemed extremely high risk or futile. Endovascular treatment approaches have been applied to a small number of these patients and data are limited to case reports and small series. The application of endovascular therapies to ascending aorta is currently limited by anatomical and technical challenges posed by the dynamic motion of the ascending aorta and the proximity of vital structures to intended landing zones (aortic valve, coronary arteries, and supra-aortic branches) and lack of specially designed endografts to address these issues. While thoracic endovascular aortic repair (TEVAR) has replaced open aortic repair for a suitable lesion in distal aortic dissection, some selected patients with type A aortic dissection at high surgical may be candidates. Hence, there is potential because, in proximal (Stanford type A) dissections, 10–30% of patients are not accepted for surgery, and 30–50% are technically amenable for TEVAR. Recent experience has shown that carefully selected patients with favorable anatomical characteristics may be subject to endovascular stent-graft treatment as a last resort with mixed results. Technical improvement is necessary to offer. satisfactory endovascular options in non-surgical candidates.

## Introduction

Acute type A aortic dissection (TAAD) is a surgical emergency with high morbidity and mortality. With early mortality varying from 17 to 26%, TAAD continues to represent a great challenge for aortic surgeons [1, 2]. Without repair, mortality can reach up to 50% within the first 48 h [3]. The primary aim of surgery is the prevention of death from aortic rupture and is mainly accomplished by excision of the proximal tear, supracoronary aortic replacement, and re-establishment of dominant flow to the distal true lumen. Although improvements in cardiopulmonary bypass, cardiac surgery techniques, and cerebral protection have been associated with better outcomes, perioperative mortality ranges from 15 to 30% particularly in the elderly with significant comorbidities [4]. In addition, neurological complications (18%) remain high. While patients’ outcome is largely determined by comorbidities and dissection-related complications, surgical mortality is high in unstable patients with pre-operative organ malperfusion.

Almost 10–30% of the patients are not accepted for surgery because they are considered unfit to undergo open repair [3] Furthermore, coma, shock secondary to pericardial tamponade, malperfusion of coronary or peripheral arteries, and stroke are important predictive factors for post-operative mortality. Recent image analysis had suggested that up to 50% of patients with proximal dissection may be technically amenable to thoracic endovascular aortic repair (TEVAR) [5, 6]. With the ultimate goal of fully catheter-based interventions to the ascending aorta, we have screened the current literature for this review.

### Favorable anatomical characteristics and technique

Achieving safe and successful endovascular access for the deployment of stent-graft devices is the most important step during TEVAR. Femoral artery access is the standard for TEVAR and is feasible in at least 70% of cases. However, due to severe tortuosity, both transapical and trans-carotid access may be explored.

An entry tear located in the center portion of the ascending aorta offers the best target for TEVAR, as the absence of an acceptable proximal landing zone is the most common exclusion criterion. Entry tears close to coronaries or aortic valves are prohibitive as is aortic regurgitation. A length of a proximal or distal aortic landing zone more than 20 mm is required in order to deploy safely. Entry tears close to the brachiocephalic artery would require complex branching/fenestration strategies; significant aortic valve regurgitation is prohibitive today, but future technology may combine a stent-graft with an integrated valve. Currently, only a limited number of choices are available regarding the type of stent-graft and delivery system as relatively large diameter and short length stent-grafts are required.

### Complications

Both, anatomic complexity and motion of ascending aorta are major obstacles to the use of endovascular technologies. Limitations arise from the short and wide shape of the ascending aorta that often curves sharply, resulting in marked disparity in the lengths of its inner and outer curves.

Complications of proximal TEVAR are neurologic as a result of brain ischemia or embolization. Excessive wire and catheter manipulation in a diseased aortic arch with risks of air embolism from deployment systems and inadvertent coverage of arch branches increase the risk of stroke. In one report, stroke occurred in three of 45 patients (6.8%)—two of which had received their stents via left common carotid artery approach [7, 8].

Another important characteristic relates to the design of stent-graft; devices without proximal bare springs are recommended to avoid compromising of the aortic valve.

Supra-aortic vessel occlusion is another important concern. If the distance between the distal landing zone of the stent-graft and the origin of the supra-aortic vessels is not adequate, the procedure can only proceed with arch vessel rerouting or bypass to generate sufficient anchoring area. Alternatively, debranching of the innominate trunk to the left common carotid artery has been used to increase the length of the distal landing zone.

Proximal type I endoleak is a common complication after stent-grafting. Rapid ventricular pacing is probably required to avoid misplacement and windsock effect and assure precise stent-graft placement [9]. The diameter of the stent-graft should be according to the previous aortic dimension (before dissection) to avoid oversizing. With the goal to re-shape the dissected ascending aorta, cover the entry tear and depressurize the false lumen (FL). It is accepted that a totally thrombosed FL is linked to better long-term survival after TEVAR [10]. In the presence of endoleaks, balloon expansion for better wall apposition or even a short extending stent or occluder can be used to seal the endoleak. Sometimes additional procedures including coils or other devices are necessary.

### Our experience

**Fig. 1:**
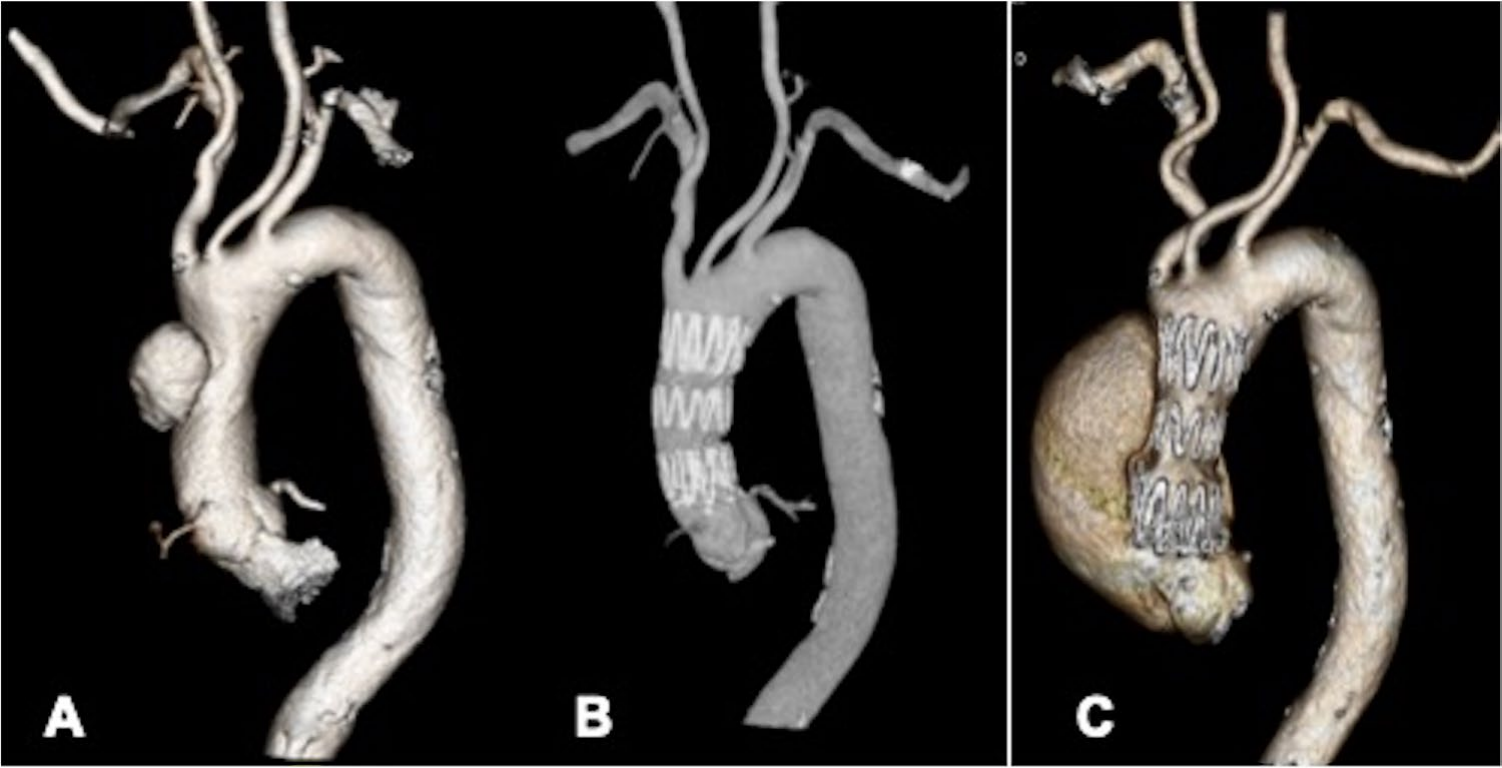
A example of late complication of thoracic endovascular aortic repair (TEVAR) in proximal aortic dissection. **A** shows a localized type A aortic dissection; **B** shows the excellent result after TEVAR with a short stent-graft; **C** shows a stent-induced re-entry tear (erosion) from the proximal contact between the crown of stent-graft and out-curve of the aorta after 6 months

Between the year 2015 and 2020, 19 patients with acute, subacute, or chronic TAAD with the proximal entry tear located between the coronaries and brachiocephalic artery were treated with TEVAR. Various stent-graft configurations were used to seal the proximal entry tear in the ascending aorta under rapid pacing. Procedural success was achieved in 17/19 patients (89.5%). There was one intra-procedural death and one minor stroke, but no additional deaths at 30 days. At 36 months, there were 4 further deaths (all from non-aortic causes). The mean survival of these 4 deceased was 23 months (range 15–36 months) [11]. Follow-up computed tomography (CT) demonstrated favorable aortic remodeling except for one case where a new proximal type I endoleak was discovered after 18 months and the patient was treated with high-risk open surgery (Fig. 1).

Procedural planning was based on contrast-enhanced, electrocardiograph-gated CT, which was evaluated with standard image processing software to select the appropriate stent-graft size. The stent-grafts used were usually ZENITH TX2 (Cook, Bloomington, Ind), GORE C-Tag (Gore Ltd, London, UK), or Relay NBS (Bolton, Barcelona, Spain). They are made of a self-expanding Nitinol stent platform covered with polyester fabric mounted onto a catheter-based delivery system.

With the patient under general anesthesia, a temporary pacing wire was placed in the right ventricle (RV) and vascular access for the TEVAR device (22–24 F) was obtained via femoral arterial cut-down. The true lumen of the aorto-ilio-femoral arterial route was navigated using a soft long hydrophilic guidewire (Terumo Medical Corp, Somerset, NJ) protruding ahead of a pigtail catheter to reach the left ventricle under fluoroscopy [12], then exchanged for a stiff wire. The stent-graft was then delivered over the stiff guidewire to its intended position, where its distal landing zone is between distal to coronary ostia and proximal to brachiocephalic artery in the ascending aorta. In this position, the distal tip of the delivery system may cross the aortic valve. For stent-graft deployment, rapid right ventricular pacing at 180 bpm was used to reduce systolic blood pressure to 50 mm Hg to avoid displacement (by windsock effect) during deployment. At the end of the procedure, the temporary pacing wire was removed, the femoral artery access site closed, and the patient extubated and transferred to the coronary care unit. Procedural success was defined as successful placement of the stent-graft in its intended position with the sealing of the entry tear.

### FL intervention to promote remodeling and thrombosis concept

The FL intervention to promote remodeling and thrombosis (FLIRT) concept relies on minimalistic, percutaneous interventions to manage the FL by inducing thrombosis, both proximally and distally, by the use of occluder devices, vascular plugs, distal stent-grafts, or coils and glue both in chronic type A and B dissection. The decision to proceed with percutaneous means to induce FL thrombosis and eventually remodeling was based on the presence of both (re)-entries and FL flow/expansion.12 All embolization and closure procedures were performed percutaneously, under local or general anesthesia, via femoral and/or brachial access. Various percutaneous techniques were utilized for FLIRT:

1. Promotion of FL thrombosis by sealing the entry tear or communication using a combination of coils and Amplatzer™ patent foramen ovale (PFO) or atrial septal defect (ASD) occluders (as short stent-graft are not suitable).
2. Retrograde catheterization of the FL via distal re-entry tears and induction of thrombosis by coils, duct occluder in addition to true lumen scaffolding with bare metal stent and/or re-entry occlusion by short stent-graft.
3. Selective application of Onyx® glue in FL and distal sealing of re-entry site by the use of a local stent-graft.

**Fig. 2:**
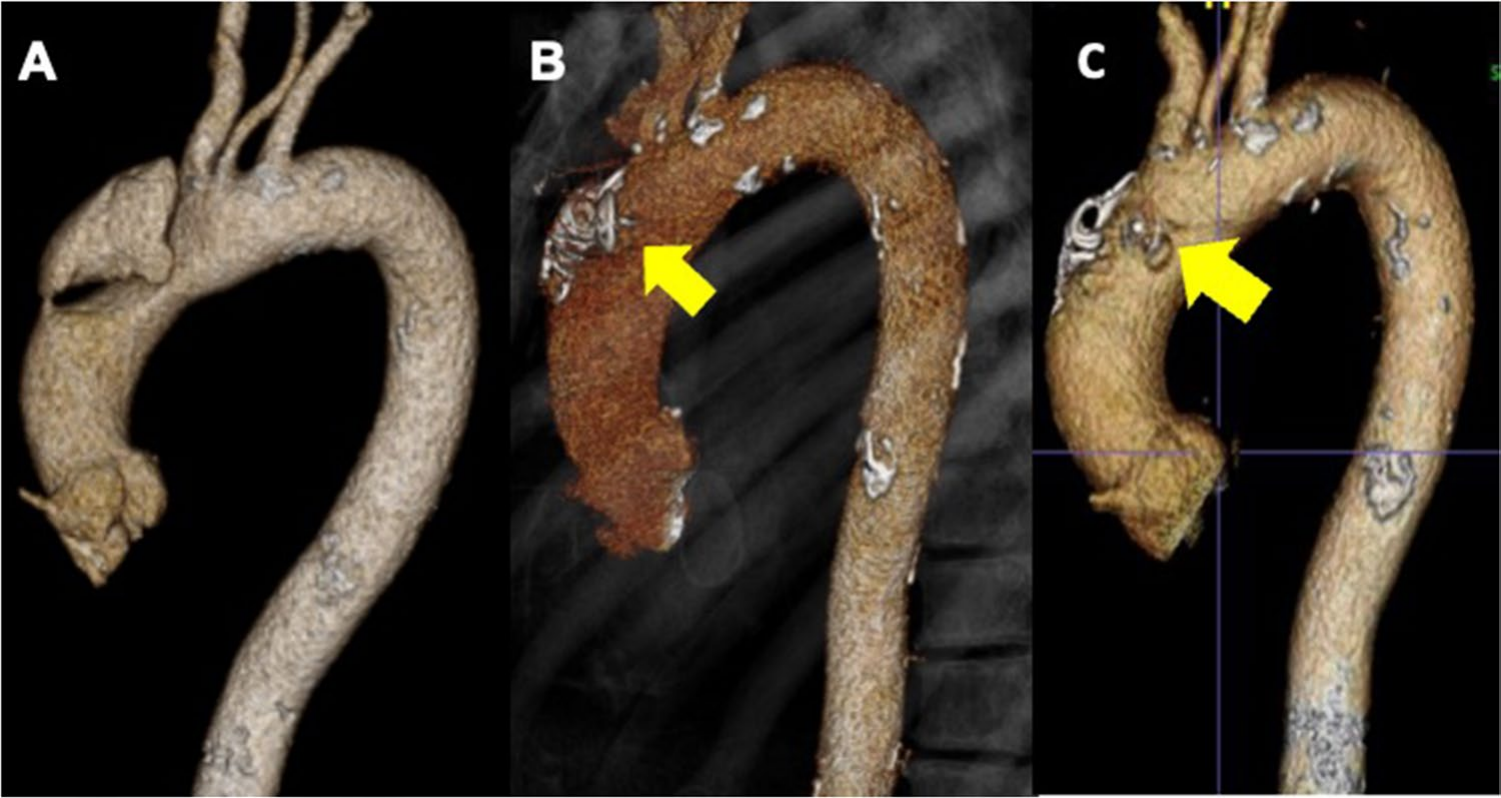
An example for false lumen intervention to promote remodeling and thrombosis (FLIRT) procedure. **A** shows a localized type A aortic dissection with an entry tear at the outer curve of the aorta just proximal to the innominate artery. **B** shows the result of endovascular treatment with coils dropped in the false lumen to promote thrombosis and a patent foramen ovale (PFO) occluder (arrow) to isolate the communication between true and false lumen (FLIRT concept). **C** shows a complete remodeling of the aorta without any complications after 2 years of procedure

In essence, the approach to managing proximal and distal dissection was fundamentally the same. Initially, a 5-F coronary diagnostic catheter was advanced into the target entry. If appropriate selective coiling was performed with spiral coils directly detached via 5F catheter (William Cook Europe, Bjæverskov, Denmark). In all type A cases, coiling was followed by definitive sealing of the proximal tear using a PFO or ASD-occluder (St. Jude Medical, Minneapolis, MN), delivered via a dedicated sheath or 8-F coronary guiding catheter (Fig. 2). Ethylene–vinyl alcohol copolymer (Onyx® glue, ev3, CA) was injected via conventional 6-F MP coronary guide catheter in 2 cases and performed in a state-of-the-art hybrid theatre offering the latest imaging technology and simultaneous transesophageal echocardiogram (TOE).

### Other reported experience

**Fig. 3:**
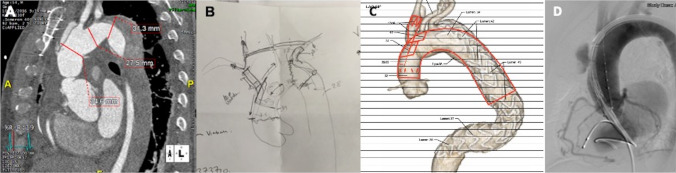
A careful assessment and planning before the procedure is the key element for a successful endovascular treatment for proximal dissection. **A** shows an extensive measurement based on gated computed tomography (CT) images; **B** shows the exploration of potential procedure in a sketch; **C** shows computational modeling to mimic the devices and outcome; **D** shows a real-world case accomplished after intensive multidisciplinary preparation

Although currently there are no multicenter clinical trials focusing on analyzing the outcome of endovascular treatment in proximal aortic disease, there are a couple of small-scale studies that reported and summarized their own experiences in this challenging and controversial technique (Fig. 3). We analyzed 20 recently published articles with available patients’ outcome dates. The number of patients enrolled in the studies ranges from 4 to 45 with a TEVAR primary success rate varies from 59.1 to 100%. The mortality rate at 1-year follow-up was noted 0 to 28.3%, while the endoleak occurred and re-intervention required after TEVAR are 0–27.3% and 0–33.3%, retrospectively (Table 1).

**Table 1:**
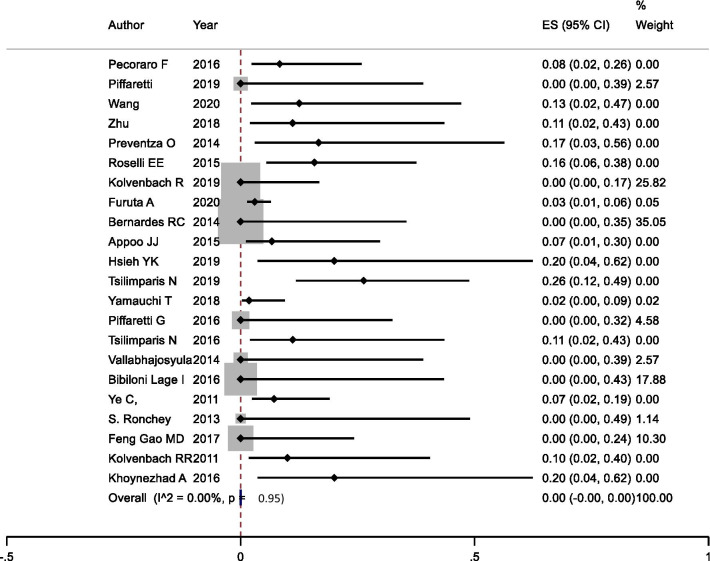
Procedural mortality—meta-analysis of TEVAR in proximal aortic dissection

## Discussion

Furthermore, the overall technical success of all studies on TEVAR in the ascending aorta was 95% and the pooled stroke rate was 7.8%. Device and delivery system limitations are likely causes of the high rate of endoleaks. Moreover, the quality of the evidence on proximal stenting of the aorta is weak. In addition to publication bias, significant variability exists, both anatomically and physiologically, regarding patient selection. These limitations should mitigate drawing any hard conclusions from feasibility studies yet. Rampant selection bias raises the one unanswered question in the consideration of endovascular therapy; e.g., what patients are suitable for such therapy? Two studies found 31.5% [13] and 36.2%5 of TAAD technically suitable for endovascular management, with a distance from entry tear to coronary ostia of 420 mm. A study by Roselli et al. [14] found this distance was 1.8 ± 0.3 cm, which may initially lead one to conclude that this fact contributed to their higher morbidity and mortality. However, complications related to coronary exclusion were responsible for only one death in this study. Additionally, the Physician-Sponsored Investigational Device Exemption (PS-IDE) protocol entails a 1-cm minimum landing zone, which was thought to be adequate for TAAD [15]. Surgeon experience and institution volume undoubtedly have a significant influence on outcomes of this technically complex approach; Table 2 summarizes the currently debated pros and cons of an endovascular approach to the ascending aorta.

**Table 2:** Endovascular management of the ascending aorta

Pros	Cons
Option for patients with extreme risk for open surgery Avoidance of thoracotomy Trans-arterial (femoral) or trans-apical access is feasible Applicable to select dissection, focal, and suture line aneurysm	Highly selected patients with suitable anatomy - Entry tear central between coronaries and brachiocephalic trunk - Proximal and distal sealing zones 20 mm - Relevant aortic valve regurgitation unsuitable - Shaggy aortic arch/high stroke risk - Rapid RV pacing is compulsory Device embolization of migration May require additional neck vessel rerouting Limited size and lengths of available stent-grafts Risk of damage to the aortic valve and left ventricle by wire of the delivery device Risk of erosion from the extensive 3-dimensional motion of the ascending aorta

Surgical mortality is reported between 10 and 25% [1, 16] today depending on the complexity of the operation and the clinical status of the patient. In experienced hands, the procedural mortality of TEVAR to the ascending aorta was 8%, comparing favorably with published endovascular mortality of 11% [17, 18]. Interestingly, most anticipated complications such as major stroke did not occur; 1 minor stroke (transient) and 1 death from guidewire perforation of the left ventricle had led to fatal tamponade in our series. The number of TEVAR procedures performed relative to all emergency surgeries for TAAD in our 3 centers is almost 2%, or 6–17% of inoperable type A cases.

### Goals of an endovascular strategy

Successful sealing of the proximal entry with no development of proximal type I endoleak is the goal and was achieved in all cases compared to 10% failure rate reported elsewhere [19] in the attempt to reshape the dissected ascending aorta and depressurize the FL [7, 20]. Once precisely deployed, the process of remodeling of the FL appears similar in both proximal and distal dissections [21, 22]. Most of our cases were DeBakey type II dissections, and even the 2 cases of type I dissection [11] proved that the therapeutic concept of closing the entry holds true in ascending aortic dissection [23, 24]. Current experience from small case series underlines the feasibility of proximal endovascular procedures over more than 10 years [6, 20, 22, 25–27]. Our series over 6 years with a mean follow-up of > 20 months is encouraging considering inoperability of those patients. In other words, it is feasible to avoid high-risk/complex surgery and apply less-traumatic interventions to obtain acceptable outcomes. The advantage of TEVAR includes the avoidance of thoracotomy, cardiopulmonary bypass, selective head perfusion, and associated surgical risks in a high-risk elderly population [28]. If the high initial 60% mortality of TAAD not accepted for surgery can be successfully lowered by TEVAR, such a less traumatic strategy may potentially become an option in a broader spectrum of patients [16].

TEVAR will not be feasible in every patient due to anatomic constraints. Currently, only limited choices are available regarding the type of stent-graft and delivery system as large diameter and short length stent-grafts are required. Existing delivery systems need to be modified with a short nosecone not to damage the aortic valve or cause left ventricular perforation. In the context of stent-graft delivery, rapid RV pacing is probably the most efficient method to avoid the windsock effect, enabling precise stent-graft placement. Transesophageal echocardiography is also useful in guiding stent-graft positioning and assessing the sealing of the entry tear [12]. It should be emphasized that a multidisciplinary team, providing surgical and interventional competence, should perform such a procedure similar to transcatheter aortic valve replacement. TEVAR in the ascending aorta can be a definitive solution for patients not accepted for surgery, or a bridging solution in case of unclear neurological scenarios (e.g., major stroke) to buy time for reconstructive surgery. It is conceivable that a combined transcatheter aortic valve and ascending graft technology may become the next technological improvement to tackle the ascending aorta.

### Ancillary procedures

In patients with organ or limb malperfusion, coverage of the proximal entry tear will usually expand the true lumen and re-perfuse ischemic organs. Patency of the FL both in ascending and descending aorta is not uncommon and is considered to herald unfavorable long-term outcomes [29, 30]. In the absence of thrombosis and remodeling, further dilatation and rupture are likely [31–33]. Thus, it appears logical to induce complete FL thrombosis to fully manage aortic dissection and improve outcomes [34] in the long-term, knowing that incomplete or partial FL thrombosis and a patent FL promote further expansion and possibly subsequent rupture (RR 2.69, *P* = 0.002) [29]. The classic endovascular approach is the occlusion of connections between true and FL by additional stent-graft coverage, or by custom-made fenestrated and branched endografts at the level of abdominal re-entries [35]. This method affords FL thrombosis throughout the dissected thoraco-abdominal aorta [36], but carries significant risks, especially for spinal cord ischemia, as many segmental arteries may be covered. Recently, alternatives were introduced including “candy-plug” and “knickerbocker” techniques, relying on FL obstruction by prohibiting distal FL backflow [37, 38]. Both techniques have drawbacks, have not been tested in larger series, and are considered more traumatic and potentially risky. Similarly, the use of multilayer stent technology would not be safe or appropriate as side branches and communications are likely to stay open [39, 40]. More interestingly, a recent “true lumen intervention” suggests tackling distal re-entry sites by small stent-grafts to promote thrombosis of the FL [41].

Conversely, the FLIRT concept uses technology applied to the FL in order to promote remodeling; it ranges from injection of embolizing material to deployment of coils, plugs alone, or in combination, as integral components of FL intervention [42, 43]. Onyx® is the most common embolizing agent and has previously been used with coils to promote FL thrombosis and seal endoleaks [44, 45].

In the ascending aorta, the FLIRT concept provides additional endovascular options. FLIRT has proven safe and feasible in 5 attempted cases of chronic type A dissection and is certainly less traumatic than other techniques including stenting of ascending aorta [43]. Closure of entry tears, restoration of single-lumen blood flow, and enhanced FL thrombosis may reap long-term benefits [11], without the risks of stent-grafting the ascending aorta (Fig. 2). Hence, the FL channel is an alternative pathway to allow a catheter to reach the tear to deploy coils and Amplatzer™ vascular occluder devices in the FL. In our series 2 Amplatzer™ Septal Occluders, 2 Amplatzer™ PFO Occluders, 1 Amplatzer™ Vascular Plug II, and 1 Amplatzer™ Duct Occluder II were used. The criteria to choose these devices were individualized to the anatomical characteristics of any given patient and to the access sites needed for different sizes of delivery systems of those devices. The sustainability of FLIRT as a concept to promote remodeling may be controversial and longer follow-up is certainly needed. Advanced interventional skills, with formal training in interventional cardiology, are also essential for safety interventions. Finally, the best timing of FLIRT as a complimentary intervention is not clear yet, but a better understanding of CT imaging may help to identify patients likely to benefit from FLIRT [46, 47].

### Technical aspects of endo-grafting the ascending aorta

Although the intimate proximity of the aortic valve and coronary ostia necessitates precise deployment of the graft specifically in the region of ascending pathology (despite the greater forward flow forces that hinder precision), smaller landing zones do not seem to explain morbidity and mortality. Still, given the complexity of the ascending aorta and the uniqueness of the local anatomy and hemodynamic forces, the ultimate quandary is one of operability based on the currently available technology. The ability of gated CT imaging to quantify intimal defects provides crucial information for deciding which patients are candidates for endovascular approaches and for procedural planning in this delicate region. The curvature in this area of the aorta is steep and the diameter and length fluctuate significantly, explaining the difficulty in the coaxial deployment of the graft from a transfemoral approach. An alternative is the transapical approach [48, 49], which is occasionally used for cardiovascular operations such as ventricular assist devices and transcatheter aortic valve replacement. The short distance to the ascending aorta can increase the accuracy of deployment in the context of a limited landing zone, but the access is quite traumatic. Immediate pericardial drainage is possible in patients developing pericardial effusion. With further technical development and investigation of various approaches, this strategy may become important. Other options include axillary and carotid cannulation as well as transseptal access from the femoral vein. Low-profile delivery systems may broaden the feasibility and success of new approaches.

From a purely technical standpoint, dissection-specific devices are necessary for better procedural outcomes. In 2012, Metcalfe et al. [20] reported the first use of a device designed explicitly for the ascending aorta in the UK (a 34-mm-diameter Zenith Ascending Dissection device; Cook Medical, Bjaeverskov, Denmark). In the USA, the first stent-graft for use in the ascending aorta is the modified COOK TX2 Thoracic Stent-graft without proximal bare metal (with a diameter up to 46 mm and a treatment length up to 90 mm), and the investigational short GORE Active Control device is used in the ongoing Ascend trial. Fenestrated, branched, bifurcate, and custom-made endografts may also play a role in the future of endovascular treatment of TAAD [50, 51]. Additionally, strategies for graft delivery must be upgraded alongside the design of arch-specific stent-grafts. Because of tortuosity encountered in traversing the iliac vessels and the arch, trackability, pushability, and torquability may be lost. Pre-curved delivery systems and specific guidewire configurations may improve coaxial deployment via the typical TEVAR transfemoral approach.

### Current technological limitations

With no devices currently approved for use in the ascending aorta, there is a significant limitation of available stent-grafts. To date, all cases have received off-label devices and have been limited to select cases in which the patient’s anatomy could be aligned with available sizing options. These sizing limitations illustrate the need for approved devices designed specifically to conform to the anatomic challenges of the ascending aorta.

Whereas femoral artery access is preferred, some delivery devices are not long enough to reach the ascending aorta; the axillary and carotid arteries are less likely to be affected by peripheral vascular disease and therefore can often provide viable access in patients with severely diseased femoral or iliac vessels [52]. Review of the literature showed no difference in outcomes with the use of a femoral vs. alternative access approach that can be safely performed in the hands of a skilled endovascular specialist sometimes with the use of a conduit as an access. Despite a cohort of exclusively high-risk patients, early outcomes appear promising in those individuals treated endovascularly for ascending aortic conditions. Many of the patients treated with ascending aortic stent-grafts were considered unfit for open surgery on the basis of their medical comorbidities. Aorta-related mortality was 5% and all-cause mortality was 14.9%, which is significantly lower than that observed for patients with type A dissections treated with open surgery, which ranges from 17 to 26% [53]. One population of patients that is poised to benefit from endovascular therapies in the ascending aorta is patients with acute TAADs. The International Registry of Acute Aortic Dissection reports that patients undergoing open surgical repair for acute type A dissections have an in-hospital mortality of 26.9%; moreover, up to 30% of patients are considered unfit candidates for open surgery on the basis of advanced age, medical comorbidities, and preference of the patient [54]. Endovascular approaches are an appealing prospect for these patients because they are minimally invasive, circumvent the need for cardiopulmonary bypass, and involve a much less extensive surgery. Whereas it remains to be seen whether these interventions will provide a long-term solution for these patients, one application of the technology is the stabilization of acutely sick patients who can later go on to have a definitive open repair.

The complication most commonly encountered in patients with ascending aortic stent-grafts is endoleak, with an overall rate of 18.2%. The overall endoleak rate encountered in TEVAR ranges from 3.6 to 8.7% and represents a significant limitation of this technology [55]. Endoleak can contribute to FL expansion in dissections and increase the risk for aortic rupture. In response, a number of promising technologies including endoanchors, branched stent-grafts, and in situ fenestration are currently being used both to prevent and to treat endoleak in the abdominal aorta and aortic arch [56–58]. As adjunctive technologies continue to develop, they may in the future help overcome issues of endoleak due to poor stent-graft deployment or inadequate landing zones in the ascending aorta.

### Limited evidence for TEVAR

With the majority of manuscripts consisting of case reports and small series, there remains a strong selection bias in the literature with regard to endovascular management of the ascending aorta; Table 1 is a summary of previously published evidence. Within the past 2 years, some larger studies have been published that help providing greater insight into emerging ascending aortic interventions. Li et al. presented one of the largest cohorts to date, with a total of 15 patients with TAAD treated endovascularly by stenting of the ascending aorta. In this cohort, long-term follow-up revealed 8 complications, including new dissection, cardiovascular ischemia, retrograde TAAD and ventricular pseudoaneurysm, pericardial effusion, kidney malperfusion, and perigraft endoleak. No deaths were reported in the follow-up period. A total of four reinterventions were documented, including branched stent-graft deployment for new dissection, coronary stenting, and one open surgical repair. Although the overall complication rate was high in these patients, close monitoring allowed early detection and successful treatment avoiding long-term mortality. In addition, these patients were originally selected to receive endovascular interventions after being deemed ineligible for open surgical repair [59] suggesting that endovascular interventions can serve as a valuable intervention for patients who are considered unfit candidates for open surgery. Nevertheless, close follow-up is needed to prevent and treat complications as these arise. Thus far, most published results are positive, but the data are based almost exclusively on single-center experiences in case reports or case series. To truly understand the outcomes associated with endovascular interventions in the ascending aorta, a prospective, randomized controlled trial should be advised and completed, ideally across multiple, high-volume aortic centers and the use of stent-graft designed specifically for the ascending aorta. With the current device limitations, this technology should be reserved for high-risk surgical candidates, particularly those denied open interventions. Interventions should be performed in select patients whose focal ascending aortic lesions can be addressed with currently available devices. In addition, these procedures should be performed by experienced endovascular specialists, and steps should be in place for open interventions when complications arise (Fig. 3).

## Summary and outlook

TEVAR in proximal aortic dissection can be performed with high technical success and acceptable morbidity and mortality in high-risk inoperable patients. Optimal aortic remodeling could be achieved by using new short-length stent-graft devices or ancillary technology like FLIRT. The current iteration of stent-graft technology however needs to be adapted to the specific challenges of the ascending aorta.
